# Calcium Spikes in Epithelium: study on *Drosophila* early embryos

**DOI:** 10.1038/srep11379

**Published:** 2015-07-22

**Authors:** Olga Markova, Sébastien Sénatore, Claire Chardès, Pierre-François Lenne

**Affiliations:** 1 Aix Marseille Université, CNRS, IBDM UMR 7288, 13288, Marseille, France

## Abstract

Calcium ion acts in nearly every aspect of cellular life. The versatility and specificity required for such a ubiquitous role is ensured by the spatio-temporal dynamics of calcium concentration variations. While calcium signal dynamics has been extensively studied in cell cultures and adult tissues, little is known about calcium activity during early tissue morphogenesis. We monitored intracellular calcium concentration in *Drosophila* gastrula and revealed single cell calcium spikes that were short-lived, rare and showed strong variability among embryos. We quantitatively described the spatio-temporal dynamics of these spikes and analyzed their potential origins and nature by introducing physical and chemical perturbations. Our data highlight the inter- and intra-tissue variability of calcium activity during tissue morphogenesis.

Variation in intracellular calcium concentration is a fundamental signaling event that controls numerous biological functions including cell division, muscle contraction, apoptosis and gene expression^1^. The universality and ubiquity of calcium signaling rely on the presence of thousands of calcium sensing proteins^2^. These proteins can be activated by changes in calcium concentration, which occur with various duration and localization inside the cells^3^.

The importance of calcium signaling in morphogenesis has been shown in several model organisms. Inhibition of calcium signaling leads to convergent extension defects in both *Xenopus*^4^ and Zebrafish^5^, and impairs epiboly progression in Zebrafish^6^ and newt^7^ as well as the formation of somites in chic k^8^. Experimentally-induced increase in calcium concentration promotes tissue elongation in *Drosophila* egg chamber^9^, increases the rate of somitogenesis in chick^8^ and Zebrafish^10^, activates gastrulation in sea urchin^11^, and triggers neural fold formation in *Ambystoma*^12^.

In the developing embryo, two patterns of calcium activity have been observed: calcium waves and spikes^13^,^14^,^15^. Calcium waves arise in a spontaneous and repetitive manner during gastrulation of Zebrafish^16^,^17^,^18^ and *Xenopus*^19^ and are sometimes accompanied by a wave of tissue contraction^4^. In *Xenopus*, such waves trigger neural induction and specific calcium-dependent genes responsible for the neuralization of the dorsal ectoderm^12^,^19^. In several biological processes, including wound healing^20^, calcium waves provide rapid communication across large cell populations^21^,^22^. Calcium spikes on the other hand are short-lasting and local events that appear stochastically in single cells or in groups of a few cells^8^,^10^,^15^,^23^,^24^. Loss- and gain-of-function analyses indicate that these spikes are associated with the Wnt signaling pathway in Zebrafish embryos^25^. A decrease in spiking activity, induced by Wnt signaling inhibition, impairs morphogenesis in the developing embryo^26^,^27^,^28^,^29^.

Intrigued by the presence of calcium spikes in early embryos, we wished to assess the feasibility of using a tractable system such as *Drosophila* which is amenable to imaging and quantitative approaches, to study these spikes in detail. To date, only two studies on calcium signaling in *Drosophila* early embryos (2–4 hours after egg lying) have been published^30^,^31^ and no evidence exists that supports the presence of calcium spikes. The universality of calcium signal patterns remained therefore questionable.

In this study, we recorded variations of the cytosolic calcium concentration in *Drosophila* early embryos *in vivo*. We show for the first time in this biological model, the presence of spontaneous calcium spikes both in single and in groups of epithelial cells. We provide a detailed analysis of the spatial and temporal dynamics of calcium spiking. Finally, we demonstrate that physical pressure induces calcium spikes. Possible biological processes affected by this signaling are discussed.

## Methods

### The GCaMP3 strain of Drosophila

GCaMP3 reporter flies were constructed using classic P-element transgenesis. The G-CaMP3 (genbank HM143847.1) sequence was cloned by PCR with the pEGFP-N1G-CaMP3 vector from Addgene (ref #22692), using the 5′ primers “ TTAATAACTAGTCAC and CATGGGTTCTCATCATCA” including the specific restriction site SpeI and the 3′ primer “TTAATAGGATCCCGCTTACTTCGCTGTCATCAT” including the specific restriction site BamHI. The polymerase taq used was Phusion High Fidelity DNA Polymerase (ref #F-534S) from Thermo Scientific. The PCR program was : 98 °C for 2 min, then 22 cycles of 98 °C for 15 sec, 64°C for 30 sec, and 72°C for 40 sec, followed by 72°C during 3 min. Digestion was achieved using fastdigest enzymes SpeI/BamHI from Thermo Scientific. The p Casper vector including the spaghetti squash promotor sequence^32^ was then digested by enzymes SpeI/BamHI from Promega. The ligation was made with a ratio insert/vector of 3/1 using 0.1 μl of ligase from Thermo Scientific. Construct transformation was achieved with electromax competent cells from Invitrogen. The transformed construct was sequenced before being sent to the company “Flyfacility” for injection into flies, using their P-element basic service. The fly strain established was then mapped and expression of the construct checked by PCR on cDNA from embryos.

### Other *Drosophila* strains

A palmitoyl-anchored *venus* construct under the nanos-GAP43::Venus promoter was used to visualize cell membranes. This strain was kindly provided by Dr. E. Mavrakis^33^.

*Care and maintenance of Drosophila and preparation of embryos* for imaging were as described in detail elsewhere^34^. In short, YW *Drosophila* flies were kept at 22 °C in plastic bottles containing a solid food base with breathable stoppers. The adult flies were transferred to cages with fresh agar plates and after 30 min, the embryos were collected. Embryos were dechorionated in bleach during 30 sec, rinsed 3 times with water, placed on soft agar and aligned manually with a needle. The glue was dissolved from packaging tape in heptane and centrifuged to eliminate undissolved particles. Glue–heptane solution was then pipetted onto the center of the coverslip by glass tube and homogeneously distributed on the coverslip the day before the experiment to allow drying. The cover slip was then put onto the embryos which subsequently stuck to it. Embryos were then desiccated for 8 min and covered with mineral halocarbon oil to prevent further drying. Any embryos that developed wrinkles in the vitelline membrane during desiccation were discarded.

### Chemicals

5,5**′**-dibromo BAPTA (tetrapotassium salt) was purchased from Invitrogen.

### Microinjection

Borosilicate glass micropipettes (GC100TF-10; Harvard apparatus) were used for injection with the Eppendorf microinjection system (pneumatic picopump; World Precision Instruments, Inc.). Syncytial-staged embryos were injected with 0.01 nl-0.1 nl of 2–5 mM calcium green dextran (CaGr 10 kDa; Invitrogen) and 2–5 mM rhodamine dextran (Rhod 10 kDa, Invitrogen) suspended in water. The injection was achieved by applying a short pressure puff as soon as the needle tip had been placed inside the syncytial cell.

### Injection in the extracellular space

Injection in the extracellular space was done using the same method as described previously^34^. In short, the same equipment as that described above was used for the injection into the syncytium. Injection into the extracellular space was achieved by piercing the embryo with a needle that was then slowly retracted up to the extracellular (perivitelline) space to position the needle tip between the plasma membrane of syncytial cells and the perivitelline membrane. Once the tip of the needle was within the vicinity of the plasma membrane, the injection puff was applied. As the distance between plasma membrane and perivitelline membrane is in the order of one to tens of micrometers (depending on the embryonic region), it was difficult to see the exact position of the needle tip. Thus 2–5 mM rhodamine was added to the solution before its injection to ensure correct localization. Only the embryos in which dye had spread uniquely in the extracellular space were used for experiments and not those in which the dye had entered into the cytosol. The localization of the injected dye was checked under the microscope with 20x magnification before each acquisition and any embryos with dye inside the cells were rejected from use in further experiments.

### Injected water solutions were

0 mM, 50 mM, 100 mM, 0.5 M and 1 M CaCl_2_; 0 mM, 2 mM, 5 mM, 10 mM and 30 mM BAPTA; and 0 mM, 2 mM, 10 mM, 50 mM and 100 mM EDTA. To all solutions, 2-5 mM rhodamine was added to mark injected embryos. The final concentrations of solutions after intracellular injection were100–1000 times smaller than the injected concentrations due to dilution. This was also the case for extracellular injections, as there are no tight junctions at early stages of development and solutions could diffuse into the yolk (injected volumes are 0.01–0.1 nl, embryo volume is 10 nl). We used EDTA and BAPTA to inject extra- and intra-cellularly, respectively, in order to adapt to the different ionic compositions of these compartments.

### Physical treatments

*Injection.* The injection of water was performed as described above. The injected volume was 0.1 nl or 1 nl.

### Transient mechanical flattening

10–20 embryos were put on soft agar and covered by a glass coverslip. To control the amount of applied pressure, the procedure was performed on a balance. A 1–10 nM force (monitored continuously by the balance) was applied by human pressing down on the 24×32 mm coverslip with the needle during 6 sec (3×2 s with a few seconds interruption between successive applications).

### Imaging

Time lapse movies were acquired using confocal spinning disc microscopes from Roper and Perkin Elmer with 20× 0.75NA W, 40× 1.2NA W and 100× 1.4NA oil objectives (Nikon). The recording plane was located in the outer epithelial layer of the *Drosophila* embryo, corresponding to the region located 2–10 μm from the apical surface. Temperature during recordings was 21°C. Time delay between frames was 1–10 sec and acquisition movies were acquired over 1 to 36 h. Fluorescence was excited by laser lines at 491 and 561 nm.

*Calcium imaging* was performed using chemical and genetic sensors.

### Chemical sensors

calcium-sensitive calcium green dextran (10 kD CaGr) and the calcium-insensitive rhodamine dextran (10 kD Rhod) were used for ratiometric calcium measurements. CaGr and Rhod were excited at 491 nm and 561 nm, respectively. Genetic sensor: The single wavelength genetic sensor GCaMP was exited by a 491 nm laser.

### Data analysis

Ratiometric calcium measurements: Image analysis was performed using Fiji. The ratio between CaGr and Rhod frames was calculated after background subtraction. The value for background intensity was taken in regions free of embryos for both wavelengths. The corresponding background was subtracted from each wavelength using Process->Math tool in Fiji. The ratio of corresponding frames of both movies was calculated using Process->Calculator Plus tool in Fiji. Spikes were experimentally defined as an increase of fluorescence intensity larger than the standard deviation of the fluorescence signal and occurring in the whole cell area.

### Statistical analysis

A two-sample t-test was used to discriminate between two groups of data and to calculate p-values. Mean values and standard deviations are shown in all figures. For the experiments using physical treatments ANOVA tests were done using the anova1 function in MatLab.

Ethics statement and data availability: This study was carried out in strict accordance with the recommendations in the Guide for the Care and Use of Laboratory Animals of the National Center of Scientific Research. No ethics approval was required for work with *Drosophila*. All our data and fly lines published in this article will be provided upon request.

## Results

### Calcium spikes in the epithelium of *Drosophila* early *embryos*

**Figure 1:**
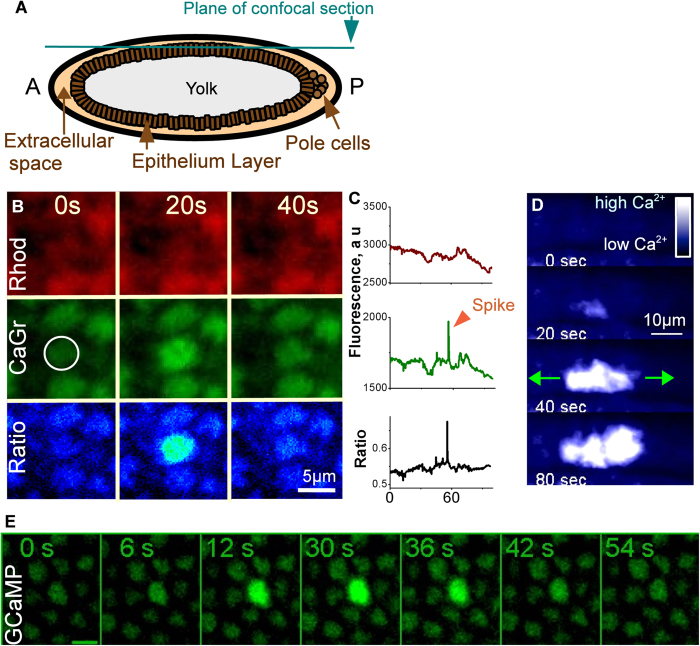
Calcium spikes in the epithelium of Drosophila embryo. **A.** Cartoon of a *Drosophila* early embryo with the location of the imaging plane indicated. **B.** Calcium spike in single cells. Upper panel: rhodamine dextran (Rhod). Middle panel: calcium green dextran (CaGr). Bottom panel: ratio of CaGr to Rhod fluorescence. **C.** Fluorescence intensity in a single spiking cell (delimited in A by a circle). **D.** A group of spiking cells. Calcium signal activated in one cell propagates to seven neighboring cells. **E.** Calcium spike in single cell recorded by GCaMP.

To monitor calcium activity in *Drosophila* embryos, we used two types of dye: calcium green dextran (CaGr), a chemical calcium sensor, and GCaMP, a genetically encoded calcium sensor. For confocal microscopy, we used a plane of focus situated at the vicinity of the apical surfaces of the epithelium layer (Fig. 1A). In combination with CaGr, we used the calcium-insensitive fluorescent reporter rhodamine dextran (Rhod) to monitor changes in fluorescence that might occur independently of changes in calcium concentration. Co-injection of CaGr and Rhod during the syncytial stage of embryos allowed the diffusion of reporters in the whole syncytial cell and thus the presence of reporters in all epithelial cells after cellularization. We monitored the variations of calcium concentrations during development by acquiring time-lapse movies from 2.5 hour after egg laying (ael) (middle of stage 5, cellularization) to 5 h ael (stage 10, end of germ band elongation). Acquisition at 1–10 sec interval between frames revealed single cell spontaneous increases in fluorescence signals of CaGr, while the Rhod signal remained constant (Fig. 1B,C). Thus, the observed changes of CaGr fluorescence reflected a genuine calcium signal. Independent recordings using GCaMP expressing embryos also revealed single-cell transient calcium increases (Fig. 1E). Both fluorescence probes agreed on the presence of calcium spikes in single cells in *Drosophila* early embryos.

Analysis of 1084 spikes (in 60 embryos) showed that 97% of calcium spikes were restricted to single cells. However, in 9 embryos, we detected 33 spikes (3% of all observed spikes) that propagated to 2–10 neighboring cells (Fig. 1D). The velocity of the calcium wave was 0.1 μm/sec (N = 15 groups of cells), 100 times slower than gap junction-mediated calcium waves (10–40 μm/sec (ref. 21)).

**Figure 2:**
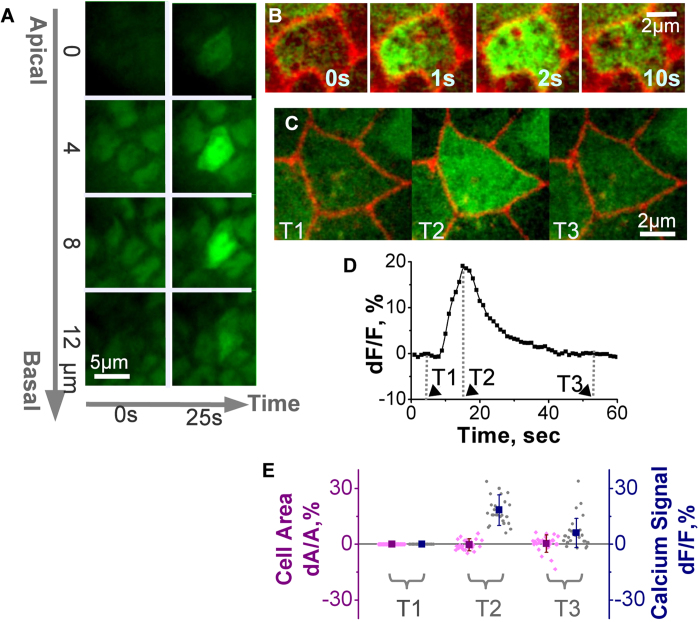
Spikes at the cellular level. **A**. Z stack of a cell before and during spiking. **B**. Propagation of calcium increase inside the cell during a spike. Red: the membrane marker nanos-GAP43::Venus; green: CaGr. **C–E.** Cell area changes during spike. **C.** Snapshots from a time-lapse movie. Red: the membrane marker nanos-GAP43::Venus; green: CaGr. Right image: before spike (T1), middle image: at the maximum of calcium increase (T2), right image: after spike (T3). **D.** Time course of a calcium spike in a single cell. Time points T1, T2 and T3 define times before, during and after spike, respectively. **E.** Changes in cell area and relative fluorescence of calcium signal (dF/F) before, during and after spike at time points T1, T2 and T3. N = 30 spikes.

In an attempt to localize the origins of the calcium signal within a cell, we imaged embryos at higher magnification and found that the spikes occurred throughout the cytoplasm of the cell (Fig. 2A). Imaging with 1 s temporal resolution acquisition showed the calcium signal spreading throughout the cell within a few seconds (Figs 2B and 1 representative example of 24 observed). The duration of calcium signal propagation inside the cell was about one second (N = 10 cells), which is much longer than the diffusion time of calcium ions in the cytoplasm (about 0.02 s for a cell of 5 μm in diameter)^35^ and in the range of the intracellular calcium waves propagated by calcium-activated calcium release^22^.

On the basis of data from several reports indicating that calcium signals lead to cell contractions in vertebrate gastrulae (Xenopus^4^, Medaca^14^), we used a membrane marker (gap43 fused to the fluorescent reporter mVenus)^33^ to analyze changes in cell shape during spikes. Analysis of 30 spikes showed no change in the shape of the cell surface either before or during spiking (Fig. 2C–E), thus ruling out the possibility that spikes trigger cell contractions.

**Figure 3:**
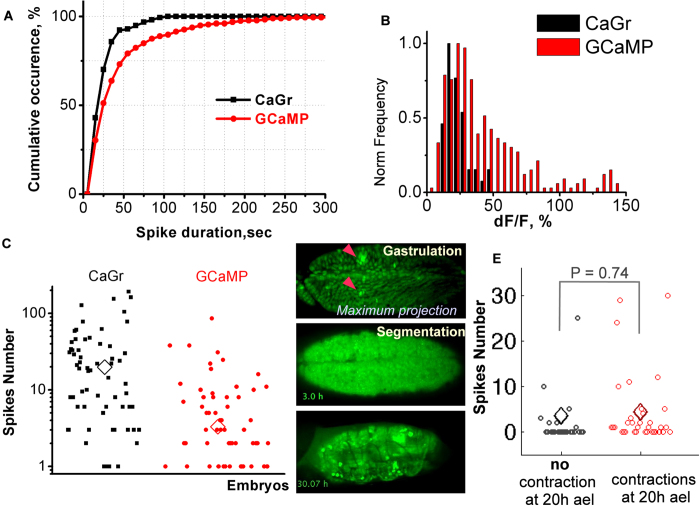
Quantification of calcium spikes characteristics. **A.** Cumulative occurrences of spike duration measured by CaGr and GCaMP. **B.** Relative fluorescence increase (dF/F) during spike. The frequency is normalized to maximum value. **C.** Variability of spiking activity. Number of spikes detected for each embryo from the middle of cellularization to germ band extension (2–4 h ael) by CaGr and GCaMP. **D.** Snapshots of a movie of a spiking embryo that passed embryogenesis, reached larva stage and successfully hatched from the egg shell (Supplementary Movie 1). Arrowheads in the upper panel indicate some spikes. In total, 32 spikes occurred during gastrulation in this embryo. **E.** Number of calcium spikes during gastulation versus the presence of contractions at 20 h ael. Mean values are indicated by diamonds. Two-sample t-test was used (P-values shown).

We then quantified the duration of spikes. In embryos loaded with CaGr (analysis of 154 spikes in 10 embryos) 50% of calcium increases were visible for less then 25 sec while in GCaMP embryos (analysis of 350 spikes in 22 embryos) 50% of spikes were visible for less then 50 sec (Fig. 3A). The mean spike duration of 26 sec and 44 sec respectively obtained with CaGr and GCaMP was significantly different, with a p value determined by t-test of 1.6e-5. For both sensors, the duration of these spikes was in the same order of magnitude as that reported in vertebrate gastrulae^10^,^19^,^23^,^29^.

We then determined the amplitude of fluorescence increase during spikes by measuring the maximum value of fluorescence increase (dF) and normalizing this to the basal fluorescent level (F), in GCaMP (N = 293 spikes in 18 embryos) and CaGr (N = 44 spikes in 3 embryos) embryos. The basal level fluorescence had a noise of 4% (Std/F). For both sensors, the amplitude distributed broadly with peaks at 20% for CaGr and 25% for GCaMP (Fig. 3B), thus showing a better signal-to-noise ratio of GCaMP than CaGr.

To quantify spiking activity, we measured the number of spikes occurring during 2–4 h ael (a period that includes the end of cellularization, mesoderm invagination and germ band elongation). Of the 97 embryos loaded with CaGr and 271 embryos expressing GCaMP (Fig. 3C), 35% and 77% respectively displayed no spikes. In spiking embryos, the number of spikes varied from 1 to 192 for CaGr embryos and from 1 to 86 for GCaMP embryos. Statistical analysis (t-test) revealed a significantly smaller mean spiking activity in GCaMP embryos (3 spikes per embryo) in comparison with CaGr-loaded embryos (20 spikes per embryo) with a p value 4e-7. As the expression level of GCaMP driven by sqh promoter is the same in all embryos while the amount of injected CaGr could vary during injection, we hypothesized that the injection procedure itself could have introduced spiking activity (for test, see later section Physical treatments influence on the spiking activity). An alternative explanation could be that the GCaMP sensor is less sensitive to variations in calcium concentration (Kd of GCaMP is 660 nM^37^ Kd of CaGr is 190 nM, Invitrogen). In any case, the two order of magnitude variability in the spiking activity between different embryos is striking.

As relatively a small number of embryos produced high spiking activity, we wondered whether these embryos managed to develop. We monitored GCaMP expressing embryo development during 48 h (at 19 °C) until hatching. Among 15 observed embryos, 5 produced 1-5 spikes and two 30 and 12 spikes respectively. The development of spiking embryos showed no abnormalities and reached larva stage and hatched. (Fig. 3D, Supplementary movie 1).

We then wondered whether calcium spiking level correlates with normal embryonic development. The course of normal embryonic development results in the appearance of peristaltic contractions during stage 17 (17–20 h ael)^38^. We thus correlated the amount of spikes produced during gastrulation and the appearance of contractions at 20 h ael. Over 57 tracked embryos we did not measure any significant difference in spiking activity in the population, which show normal contractions at 20 h ael and the population, which does not show contractions (Fig. 3E). However the absence of a straightforward correlation between normal embryonic development as revealed by these contractions and the level of spiking does not exclude other possible roles of calcium spikes during development^15^.

### Calcium activity is required for embryo development

To address whether the endogenous calcium activity (including spiking) is required for tissue movement, we blocked calcium activity before gastrulation by injecting calcium chelator EDTA (0-100 mM) or BAPTA (0–30 mM) into the embryos. We then recorded their development over a period of 4 h.

**Figure 4:**
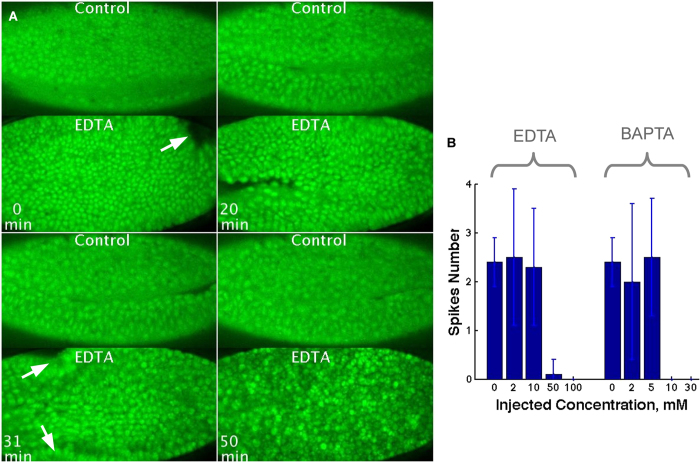
Effect of the dampening of calcium activity on the calcium spiking and embryonic development. Snapshots from movie. **A.** Upper panels: control embryos injected with water solution. Bottom panels: 100 mM of EDTA injected extracellularly at the end of cellularization and embryos imaged shortly after injection. Mesoderm invagination and further development failed in EDTA injected embyos. Calcium concentration is reported by CaGr. **B.** Quantification of the number of spikes produced during gastrulation in the embryos injected with different concentrations of EDTA. Mean values and standard deviations are shown on the graph. P-value of ANOVA test is p < 0.0001.

We first injected EDTA in the space between embryo and its envelope (extracellular space, Fig. 1A, see methods for details). The injection of 0 mM (water solution), 2 mM and 10 mM of EDTA into the extracellular space did not change embryonic development. However embryos injected with 50 mM or 100 mM of EDTA did not manage to develop. Figure.4A and Supplementary movie 2 show control embryos undergoing normal development: mesoderm invagination advances progressively in time, the region of invagination is a continuous line traversing the whole embryo along the anterior-posterior axis. After invagination cells have similar level of calcium. In embryos injected with EDTA at high concentration (50 and 100 mM), numerous defects are observed: only a fraction of mesodermal cells start to invaginate, but fail; the furrow is not straight and does not close (Fig. 4A and movie 2). Also abnormal contractions spread along the embryo. Cells become round, move chaotically and eventually the tissue collapses. At high EDTA concentration (50 and 100 mM), the number of spikes is significantly reduced during gastrulation as compared to control embryos and embryos injected with EDTA at low concentration (2 or 10 mM); ANOVA p-value < 0.0001 (Fig. 4B). This provides evidence that calcium spiking is required for normal development.

In the next series of experiments, we intracellularly injected 0–30 mM BAPTA in the syncytium before the end of cellularization, thus allowing the injected solution to diffuse to newly formed cells. Injection of 0 mM, 2 mM or 5 mM BAPTA did not perturb embryo development and spiking was not abolished (Fig. 4B), while injection of 10 mM or 30 mM BAPTA induced embryo collapse after the onset of tissue movement (data not shown). No spiking activity was recorded in these embryos (Fig. 4B). Thus, we conclude that buffering calcium activity (including spiking) at the onset of gastrulation affects early development and prevents gastrulation.

### Spatial pattern of spikes

**Figure 5:**
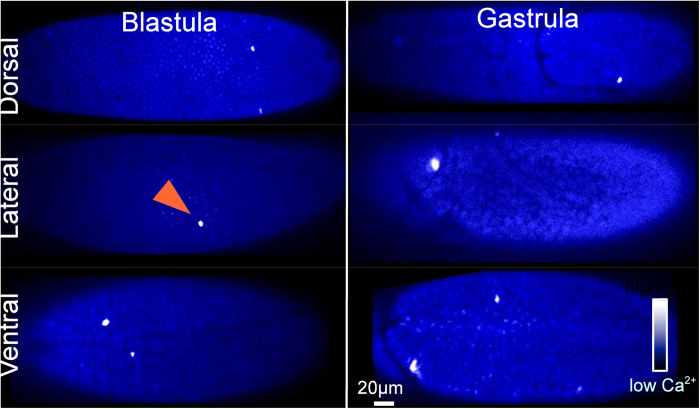
Occurrence of spikes in different regions of *Drosophila* embryo. Snapshots showing spikes in *Drosophila* blastula (left panel, 2.5-3 ael) and gastrula (right panel, 3-5 ael). Arrowhead marks a spiking cell. Measurements made using CaGr sensor.

To determine whether spiking occurs in a specific tissue precursor, we recorded calcium activity in dorsal (amnioserosa precursors), ventral (mesoderm precursors) and lateral (ectoderm and neurogenic ectoderm precursors) regions of the embryo using CaGr and GCaMP sensors (Fig. 5). With CaGr-loading, spikes occurred at ventral regions in 15 out of the 19 embryos recorded at this region, at the lateral regions in 13 out of 26, and at dorsal regions in 25 out of 30. In GCaMP-expressing embryos, spikes occurred at ventral regions in 3 out of the 9 embryos recorded in this region, at lateral regions in 11 out of 18, and at dorsal regions in 5 out of 15. These results indicate that spiking is not restricted to specific tissue precursors and precursors of different cell types are capable of spiking.

**Figure 6:**
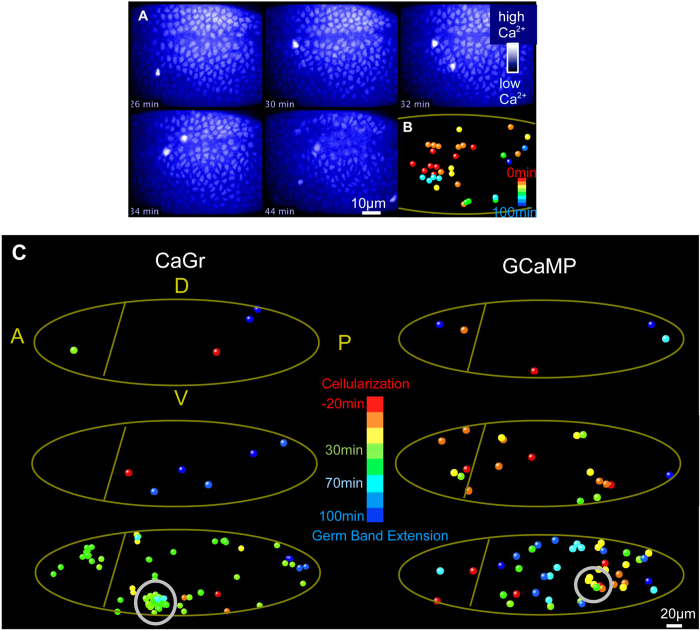
Spatial localization of spikes. **A.** Snapshots from a time-lapse movie of an embryo that underwent gastrulation, ventral view (Supplementary Movie 3). **B.** Diagram shows temporal and spatial localization of every signal detected during gastrulation of the embryo shown in A. The color code represents the temporal occurrence of spikes starting from red for the end of cellularization to blue for later stages of gastrulation. **C.** Diagrams showing temporal and spatial location of every spike detected during gastrulation in 6 embryos. Lateral regions of embryos were recorded. A, P, D and V denote anterior, posterior, dorsal and ventral regions, respectively. The color code represents the temporal occurrence of spikes from red for the end of cellularization to blue for late gastrulation. CaGr-injected embryos are on the left panel and GCaMP expressing embryos are on the right panel.

To determine whether spikes are spatially patterned, we assessed the location of each spiking cell during the whole period of gastrulation and built a spiking diagram containing both spatial and temporal information for every spike detected (Fig. 6A,B, Supplementary movie 3). The total number of recorded embryos with spikes was 66. Fig. 6C shows the embryos with different spiking activity. Spikes occurred spontaneously in different cells all over the embryo. The localization of spiking cells showed no regular pattern and the spikes did not appear to localize to any particular region of the embryo. However, in some regions, the spikes were denser than in others (Fig. 6C, circle, bottom left panel). Also, in many cases the spikes occurred in the same location but at different time points (Fig. 6, circle, bottom right panel). Altogether these data suggest the presence of a random component that determines the position of spiking cells in the embryo.

### Temporal pattern of spikes

**Figure 7:**
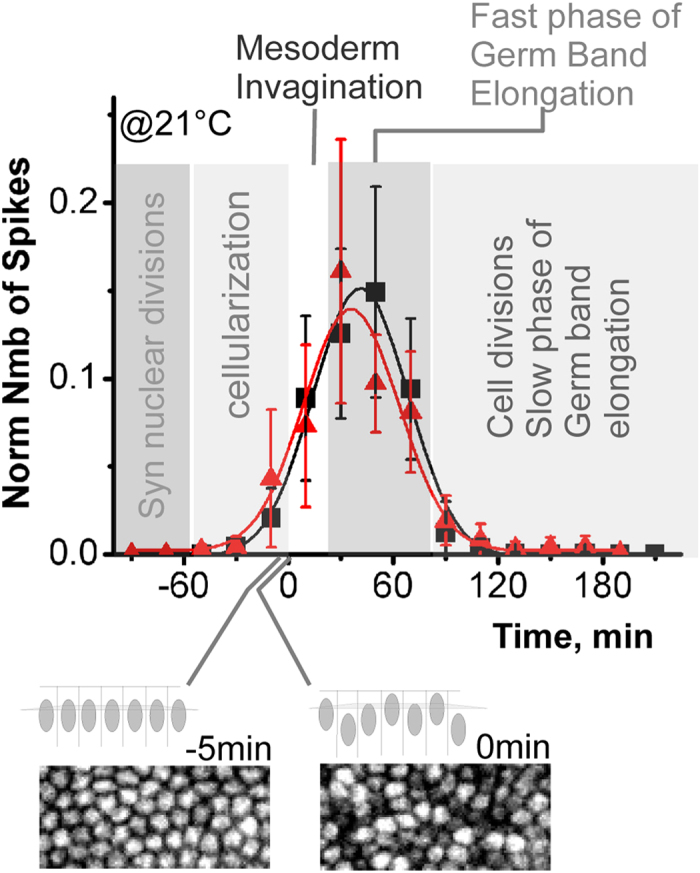
Temporal pattern of spiking activity. Zero time point corresponds to the onset of gastrulation determined as the moment when the nucleus lost its common baso-lateral position. (Schematic below shows a lateral view of nuclei along the apico-basal axis before and at the onset of gastrulation. Fluorescence images of CaGr showing corresponding snapshots). The lines are gaussian fits. For each sensor, data are averaged over 8 CaGr-injected and 8 GCaMP-expressing embryos that produced more than 20 spikes during gastrulation. Error bars are standard deviations.

To determine the temporal patterns of spiking activity, we recorded CaGr (N = 8) and GCaMP (N = 8) embryos for 5 h. We used the onset of global tissue movement as the reference time point zero (Fig. 7). The first calcium spikes started at the end of cellularization. The frequency of spikes then increased as the embryos advanced through gastrulation. The spiking activity lasted about one hour and a half and then decreased towards zero at the end of interphase 14 (Fig. 7). The peak of spiking activity, determined by gaussian fitting of the spiking profile, occurred at 39 min and 31 min after the onset of tissue movement with a sigma (half width) equal to 53 min and 54 min in CaGr embryos and GCaMP respectively. Statistical analysis using t-test found no significant difference (p = 0.1) between the mean values of these profiles. Thus our two independent measurements made with two different calcium sensors are consistent and indicate that spiking activity is regulated during development and that the spikes are mostly present during periods of mesoderm invagination and the fast phase of germ band elongation.

As the basal calcium level was reported to increase during gastrulation and decrease afterwards in different organisms including *Drosophila*^16^,^19^,^30^, we hypothesized that the temporal changes in spiking activity might be due to changes in the basal calcium levels. We thus determined whether changes in calcium levels change spiking frequency.

### Spike activation by calcium

**Figure 8:**
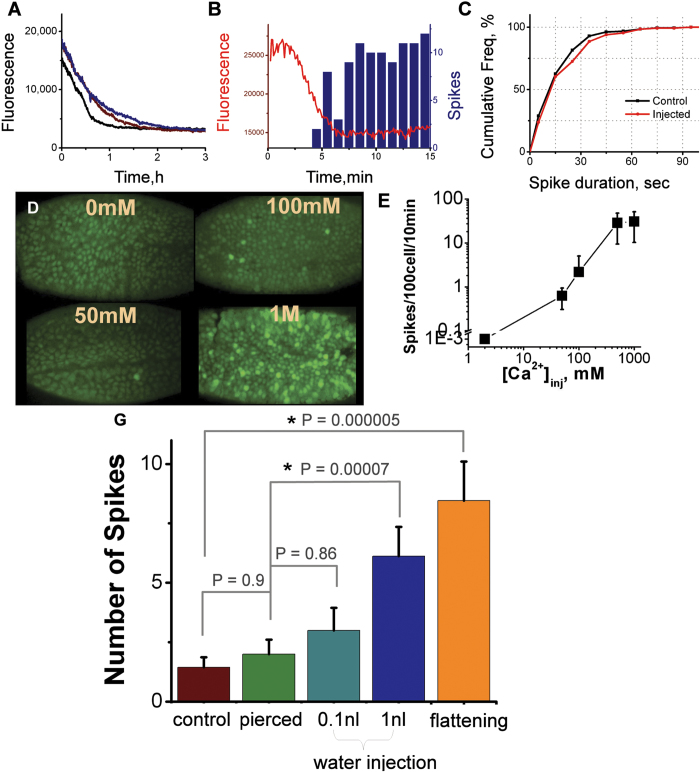
Physical and chemical treatments. **A–E.** Activation of spiking activity by extracellular calcium. **A.** Recovery to basal calcium levels after 1 M injection of CaCl_2_. Time zero corresponds to the onset of recording 20 min after injection. **B.** Appearance of spiking activity after CaCl_2_ injection. Red line shows basal calcium level recorded from the non-spiking cells. Blue bars show the number of spikes. Time zero corresponds to the onset of the recording 20 min after injection. **C.** Cumulative duration of endogenous and injection-induced spikes. **D.** Snapshots of spiking embryos injected with different concentrations of CaCl_2_ (Supplementary Movie 4). **E.** Spiking activity as a function of the concentration of injected CaCl_2_. **G.** Dependence of spiking activity on physical treatment and embryo viability. Number of spikes produced per embryo during gastrulation upon various treatments (GCaMP embryos). Two-sample t-test and ANOVA tests were used. P-values for t-tests are shown on the graphs, stars indicate when distributions are significantly different. P-value for ANOVA test is p < 0.0005.

To modify calcium levels we injected CaCl_2_ extracellularly. 1 M CaCl_2_ was injected into the extracellular space between the plasma membrane of the cells and the perivitelline membrane. We estimated that the final concentration of injected solutions was less than 10 mM due to dye dilution in the volume of the embryo and due to the action of calcium buffering systems. Extracellular injection of CaCl_2_ resulted in a transient increase in intracellular calcium concentration. This was restored to basal level 10 min–1 h after injection (Fig. 8A,B) at which point abundant spiking started in all injected embryos. Spikes occurred in a spontaneous manner in cells with even distribution throughout the embryo (Fig. 8B,D, Supplementary movie 4).

To evaluate any dose-dependent response of spiking to extracellular calcium concentration, we injected 0–1 M CaCl_2_ solution (where 0 M CaCl_2_ is a water injection). We found a correlation between the injected concentration of CaCl_2_ and the number of spiking events (Fig. 8D,E, Supplementary movie 2). The duration of spikes activated by calcium injection (N = 135 spikes in 6 embryos with CaGr as reporter) was not significantly different from non-injected embryos (analysis of 154 spikes in 10 embryos with CaGr as reporter) (t-test p = 0.8) (Fig. 8C). The extracellular injection of CaCl thus induced calcium spiking.

### Influence of physical treatment on the spiking activity

We wished to test the hypothesis that injection procedure influences spiking activity. The injection is an experimental procedure routinely used for studies on *Drosophila*, *Xenopus* and Zebrafish embryos. Nevertheless, it is an invasive method that could perturb calcium homeostasis. We hypothesized that damage induced by injection could cause spiking. Indeed, embryos injected with CaGr did display more spikes than non-injected GCaMP embryos (Fig. 3C). To clarify this point, we used GCaMP embryos. We first pricked the embryos with the injection pipette without injecting any solution (N = 20 embryos). The number of spikes did not increase significantly by comparison to the control non-injected embryos (N = 36 embryos). P value determined from t-test was p = 0.9 (Fig. 8F). We then injected different amounts of water into the embryos and compared the spiking activity of the injected and pierced embryos. Injection of 0.1 nl of water increased the number of spikes (from 2.1 ± 0.6 in pierced to 3 ± 1 in 10 injected embryos). However, the distributions were not significantly different (p value of t-test was p = 0.86). The 1 nl water injection increases the number of spikes several times in comparison with non-injected embryos (from 2.1 ± 0.6 in pierced to 6 ± 2 in 16 injected embryos). These distributions were significantly different with p values determined from t-test p = 0.00007. We then used ANOVA statistical analysis to compare these four series of experiments that suggested that at least one sample mean is significantly different for the other sample means with significance level p < 0.0005. Thus we concluded that the injection procedure could be responsible for the activation of spiking and that the amount of injected volume affects the number of spikes.

Preparation of the embryos for imaging requires their physical manipulation. Yet, these manipulations may disturb the embryos and the amount of disturbance could vary from embryo to embryo. In particular during preparation, the embryos undergo small, transient pressure when glued to the glass. To investigate how this pressure influences the spiking, we applied a transient pressure to the embryos that slightly flattened them for a few seconds. Spiking activity significantly increased in the embryos that were flattened (N = 21 embryos, p value determined by t-test p = 5e-6) (Fig. 8F). Thus we conclude that transient physical pressure leads to spiking activity.

## Discussion

Here we have described for the first time short, transient and spontaneous increases in cytosolic calcium concentrations (calcium spikes) in single cells and groups of a few cells in *Drosophila* early embryos. We then characterized the spatio-temporal aspects of these events and assessed how they can be activated using chemical and physical treatments.

Transient calcium increases have previously been observed in early embryos of vertebrates, such as Zebrafish^10^,^23^, *Xenopus*^19^ and Medaka^14^. In Zebrafish, the Wnt pathway activates calcium activity by the activation of InsP3 through frizzled and PLC^25^,^26^,^27^,^28^. The InsP3 is activated by increased calcium concentration and is a trigger in calcium-activated calcium release^44^. Recently, the presence of short single-cell calcium increases have been reported in late embryos of *Drosophila* during dorsal closure^40^. Whether the calcium transients observed in vertebrates and in late and here-demonstrated early embryos of *Drosophila* have the same physiological role and molecular mechanisms remains to be determined. Evidence for these events being related is provided by their temporal characteristics; indeed, the spike duration described in this work matches well with those observed in vertebrates^15^. Also, similarly to Zebrafish, increased calcium concentration in our work induced increases in calcium spikes frequency. However, the spike variability among embryos we described was not observed in *Xenopus* during neural induction^19^, yet was present in *Xenopus* during convergent-extension tissue movements^4^. Thus, although we have referred to the transient calcium increases observed in this work as ‘spikes’, a term used to describe calcium events in Zebrafish, *Xenopus* and late *Drosophila*, we stress that these phenomena may have different molecular pathways and physiological roles from one organism to another.

We showed that an increase in spike frequency occurs during the period of fast tissue remodeling events such as the fast phase of germ band convergent-extension and mesoderm invaginations known to be guided by increasing anisotropy in cell forces and cell shape changes^41^. Whether the spikes contribute to the convergent-extension tissue movements and cell shape in *Drosophila* remains to be elucidated. In vertebrate models, spikes have been proposed to play a direct role in the control of convergent-extension tissue movements^4^,^5^ and cell shape changes that result in apical-to-basolateral thinning of the zebrafish enveloping layer of cells^29^.

Additionally, our results show that spikes are activated by transient deformations of the embryo. The observed increase in spike frequency during the period of increased cellular tensions might indicate the activation of a particular signal transduction pathway, in particular a mechanosensitive pathway. Morphogenetic movements are closely regulated by the expression of developmental genes. The associated mechanical deformations have in turn been shown to actively participate in the signaling cascades regulating developmental gene expression. In particular in early embryos of *Drosophila*, mechanical deformation appears to induce mesoderm invagination by promoting Fog-dependent signaling through the inhibition of Fog endocytosis^42^ and upregulation of Twist in stomodeal cells^43^. Also, transient 10% uniaxial lateral deformation induces the ectopic expression of Twist around the entire dorsal-ventral axis and results in the ventralization of the embryo^44^.

The classical view is that cells from the same tissue behave similarly. However, we observed only a small number of cells in the tissue produce spikes. It is likely that at the cell level, spikes are activated in response to local variations in cell component concentrations. Thus, calcium spikes highlight intratissue cell-to-cell differences. Cell-to-cell variability is an emerging field of study, which is fostered by advances in experimental approaches at the single cell level^45^. Such variability is involved in stem-cell differentiation^45^ and cancer induction^46^. The variability of calcium events observed in this work provides a new example of intratissue cell-to-cell differences and suggests that in different cells of the same embryo, downstream pathways might be activated in some cells but not in their neighbors.

Calcium homeostasis is vitally important for the proper functioning of cells and organisms and is maintained by numerous feedback mechanisms. Interestingly, our results show that both a transient increase (calcium injection) and transient decrease (1 nl water injection) in basal calcium concentration promote spiking. Additionally the stochasticity, rareness, and the correlation of calcium activity with the physical and chemical stress, suggest that calcium spikes represent failures of calcium homeostasis mechanisms.

In summary, our work provides an *in vivo* model and a quantitative basis to study the maintenance of calcium homeostasis and the role of calcium activity during early embryonic development and tissue remodeling.

## Additional Information

**How to cite this article**: Markova, O. *et al.* Calcium Spikes in Epithelium: study on *Drosophila* early embryos. *Sci. Rep.* **5**, 11379; doi: 10.1038/srep11379 (2015).

## Supplementary Material

Supplementary Information

Supplementary Movie 1

Supplementary Movie 2

Supplementary Movie 3

Supplementary Movie 4

Supplementary Movie 5
