# Supplementary material for: Calcium Spikes in Epithelium: study on Drosophila early embryos
Source: Sci Rep. 2015 Jul 22;5:11379. doi: 10.1038/srep11379 (PMC4510484; doi:10.1038/srep11379)
Supplement: Supplementary Information [file srep11379-s1.pdf]

Calcium Spikes in Epithelium: study on *Drosophila* early embryos

Olga Markova, Sébastien Sénatore, Claire Chardès and Pierre-François Lenne

Aix Marseille Université, CNRS, IBDM UMR 7288, 13288, Marseille, France

Supplementary movies

Supplementary movies

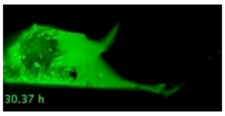

Movie. 1. Development of spiking embryo. 30 hours timelapse covers embryo development from cellularization (stage 5) till hatching (stage17, larva). The calcium spikes are visible during the gastrulation. Development proceeds normally. 20x objective lens.

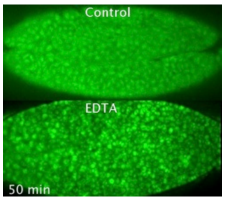

Movie. 2. Damping of calcium activity. Upper movie: control non treated embryo. Bottom movie: 100mM of EDTA was injected extracellularly at the end of cellularization. Embryos recording starts 20min after injection. 20x objective lens. Recording duration is 50min, delay between frames is 20sec. Calcium concentration is reported by CaGr.

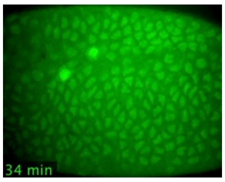

Movie. 3. Calcium spikes in the ventral regions of *Drosophila* during mesoderm invagination. Example of highly spiking embryo. Single cells and groups of few cells increase their calcium for tens of seconds. Calcium concentration is reported by CaGr. Movie duration is 100 min, delay between frames is 2 min. Recording is done with 40X objective lens.

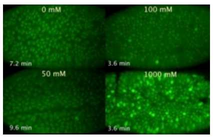

Movie. 4. Calcium activation of spiking. CaCl2 was injected extracellularly just before recordings at different concentrations (shown in the fig. 6). Calcium concentration is reported by CaGr. 20x objective lens.

Movie. 5. Calcium spikes in the embryo used for analysis in Fig.6 B.
